# 

**Published:** 1946

**Authors:** 


					Vol. LXm. No. 227.
AUTUMN, 1946.
mm
The Bristol
Medico-Chirurgical Journal
m
?4f; m
?A" . *
A: ' ? L . ?
PUBLISHED UNDEB THE AUSPICES OF v
THE BRISTOL MEDICO-CHIRURGICAL SOCIETY.
. : . ' * ,**V ?' * " '
Editor: J. A. NIXON, C.M.G., M.D., F.R.C.P.
WITH WHOM ABE ASSOCIATED
A. RENDLE SHORT, M.D., B.S., B.Sc., FJR.C.S.
?
W. MELVILLE CAPPER, M.B., B.S., F.R.C.S.
' 8|. . . <'**1
GI E. F. SUTTON, M.C., M.D., B.S., M.R.C.P.
I&feg ? m ' ?g? j ?8 .-r* ' '. .'By ?, ? E
E. WATSON-WILLIAMS, M.C., Ch.M., F.R.C.S., 2?d?or.
W&CwusIIrXkz f-jyHjf'fWiEaK!
TWU ?Tf?
.
?JflP NWfl&s
BRISTOL:
J. W. ARROWSMITH LTD., QUAY STREET & SMALL STREET
Price Four Shillings net.

				

